# Epigenetic effects of Bisphenol A on granulosa cells of mouse follicles during in vitro culture: An experimental study

**DOI:** 10.18502/ijrm.v19i2.8471

**Published:** 2021-02-21

**Authors:** Aylin Jamali Khaghani, Parisa Farrokh, Saeed Zavareh

**Affiliations:** ^1^School of Biology, Damghan University, Damghan, Iran.; ^2^Institute of Biological Sciences, Damghan University, Damghan, Iran.

**Keywords:** Bisphenol A, DNA methylation, Granulosa cells.

## Abstract

**Background:**

Bisphenol A (BPA), a synthetic endocrine-disrupting chemical, is a reproductive toxicant. Granulosa cells have significant roles in follicle development, and KIT ligand (KITL) and Anti-Müllerian hormone (AMH) are essential biomolecules produced by them during folliculogenesis.

**Objective:**

Due to the widespread use of BPA and its potential epigenetic effects, this study examined the impact of BPA on promoter methylation of *amh* and *kitl *genes in mouse granulosa cells.

**Materials and Methods:**

Preantral follicles were isolated from ovaries of immature mice and cultured for eight days. Then, follicles were treated with 50 and 100 μM of BPA, and 0.01% (v/v) ethanol for 24 and 72 hr. Growth and degeneration of follicles and antrum formation were analyzed. The granulosa cells were isolated mechanically, and their extracted DNA was treated with sodium bisulfite. The promoter regions of the *amh* and *kitl* were analyzed with PCR and sequencing.

**Results:**

BPA did not change follicle survival and antrum formation significantly (p = 0.41). However, the culture in the presence of 100 μM BPA had an inhibitory effect on growth. Before BPA treatment, the CpG of the *kitl* and *amh* promoters were unmethylated and partially methylated, respectively. While the percent of 5mC in the *amh* promoter reduced at 100 μM of BPA, it did not alter the *kitl* promoter methylation.

**Conclusion:**

BPA at higher concentrations has an inhibitory effect on follicle growth. Moreover, it seems that the epigenetic impact of BPA restricts to the demethylation of CpG sites.

## 1. Introduction

Xenobiotic endocrine-disrupting chemicals by disrupting hormone metabolism or hormone signaling pathways develop reproductive problems, hormone-related cancers, obesity, diabetes, thyroid, and nervous system disruptions (1, 2). Bisphenol A (BPA) (2,2-bis [4-hydroxyphenyl] propane) is one of the synthetic endocrine-disrupting chemicals which is widely used in polycarbonate plastic and epoxy resin manufacture (3, 4). Polycarbonate plastics are used for food and drink packaging, dental polymers (3), eye lenses, sports equipment, electronic materials, and flame retardants (5). The BPA may be absorbed by everyone through feeding, inhalation, and dermal contact (4). Broad environmental exposure of humans with products containing BPA and the finding of BPA in various biological fluids of the human body (3), including urine, milk, saliva, blood serum, amniotic fluid, and follicular fluid (6, 7), has increased concerns about its consequences on human health in recent years (3).

According to animal studies, BPA is a reproductive toxicant, and its adverse influence on gamete quality, pregnancy, and embryo development has been declared (5, 6). However, these results widely depend on the time, dose, and mode of BPA exposure (6). Epigenetic toxicity of the BPA has been reported in several studies (6, 8, 9). It seems that epigenetic modification and gene expression alteration are the main mechanisms of BPA action (5). Cellular processes, like genomic imprinting, X-chromosome inactivation, genome stability, and gene expression, are influenced by epigenetic mechanisms (10).

Granulosa cells are the most influential cells in the follicle and oocyte development is highly dependent on them (11, 12). Granulosa cells contribute to steroidogenesis, production of vascular endothelial growth factor, and oocyte nourishment (13). Granulosa cells and oocytes have bi-directional interactions that are necessary for their growth and differentiation (11, 14). One of the well-studied communications is KIT ligand (KITL) and KIT receptor. KITL is produced by granulosa cells in preantral and antral follicles, while oocytes express KIT receptor (14). The KIT–KITL interaction is essential for oocyte growth, antral cavity formation, and differentiation of theca cells (15). Anti-Müllerian hormone (AMH), a member of the transforming growth factor β superfamily, is another essential molecule that is produced by granulosa cells during preantral and small antral follicle stages. AMH plays a significant role in controlling primordial follicle pool usage both in humans and rodents (16, 17).

Because of the large-scale production of the BPA and increasing pieces of evidence which support the positive correlation of the BPA and female infertility (4, 6), understanding the various aspects of its function is worthwhile. Although some previous studies have indicated the effect of BPA exposure on the survival and function of follicle (6, 18-20) and granulosa (13, 21), no one has described the epigenetic impact of BPA exposure on the granulosa cells. Therefore, this study aimed to explore the impact of different concentrations of BPA on mouse follicular development and promoter methylation of *amh* and *kitl *genes in granulosa cells during in vitro culture.

## 2. Materials and Methods

### Animals

In this experimental study, NMRI (Naval Medical Research Institute) mice (including 15 females and 5 males) were purchased from the Pasteur Institute of Iran. The mice were kept and bred under standard laboratory conditions according to the National Institutes of Health Guide (22). Female offspring were used for next step experiment.

### Isolation and in vitro culture of follicles 

After sacrificing the 14-16-days old female mice (n = 15) by cervical dislocation, their ovaries were removed and incubated into follicle isolation medium at 37°C for 30 min. The medium was α-MEM (Gibco, England) supplemented with 25 mM HEPES, 2.2 g/l sodium bicarbonate, 10% fetal bovine serum, 75 μg/ml streptomycin, and 100 IU/ml penicillin (all of them from Sigma-Aldrich, Germany). Preantral follicles were isolated using a sterile 29-gauge needle under a stereomicroscope (Nikon, Japan) at 25× magnification. The 140–160 μm preantral follicles with normal oocyte and several layers of granulosa cells were selected under an inverted microscope with 100× magnification (Nikon, Japan). The follicles were cultured in 20 μl droplets of the follicle growth medium, under mineral oil in Petri dishes at an incubator setting of 5% CO${}_{2}$, 96% humidity, and 37°C. The basic follicle growth medium was α-MEM containing 2.2 g/l sodium bicarbonate, 0.23 mM sodium pyruvate, 5% fetal bovine serum, 75 μg/ml streptomycin, 100 IU/ml penicillin, 100 mIU/ml recombinant human follicle-stimulating hormone, 20 ng/ml recombinant epidermal growth factor (all of them from Sigma-Aldrich, Germany), and 1% Insulin-Transferrin-Selenium (Gibco, England) (23). Half of the culture was replaced by a fresh medium every 48 hr for eight days. After that, the follicles were randomly divided into six groups (with at least 15 follicles in each one) and treated as follows: (1) 50 μM of BPA (Sigma-Aldrich, Germany, purity ≥ 99%) for 24 hr; (2) 50 μM of BPA for 72 hr; (3) 100 μM of BPA for 24 hr; (4) 100 μM of BPA for 72 hr; (5) 0.01% (v/v) ethanol (solvent control) for 24 hr; and (6) 0.01% (v/v) ethanol for 72 hr. While BPA was dissolved in absolute ethanol (MERCK, Germany), the final concentration of 0.01% (v/v) ethanol was used as solvent controls.

Morphological changes of the follicles, including degeneration and antral cavity formation, were assayed during the culture time. Follicle diameter was also measured by averaging two perpendicular axis with a calibrated ocular micrometer under the inverted microscope with 100× magnification (Nikon, Japan). All experiment procedures were replicated thrice.

### Granulosa cell preparation

Granulosa cells were isolated from the follicles through pipetting by a sterile 26-gauge needle (13) under a stereomicroscope at 25× magnification (Nikon, Japan). The granulosa cells were released in PBS and centrifuged at 1000 × g for 5 min to pellet the cells.

### DNA extraction and bisulfite treatment

The genomic DNA of granulosa cells obtained from 30 follicles was extracted using a micro DNA isolation kit (Kawsar Biotech Company, K1138) following the manufacturer's instructions. After checking the quality and quantity of the extracted DNA, 500 ng of DNA was treated with sodium bisulfite using EpiJET Bisulfite Conversion Kit (Thermo Scientific, #K1461). The procedure was performed according to the manufacturer's instructions, and for maximum efficiency (> 99%); the following thermal protocol was applied: 98°C for 10 min and 60°C for 150 min. Bisulfite converts only unmethylated cytosines to uracil, while 5-methyl cytosines remain unchanged.

### PCR reaction and sequencing

The promoter regions of the *kitl *(NM_013598) and *amh *(NM_007445) genes, 1 kb upstream of the transcription start sites, were extracted using the UCSC Table Browser tool (http://genome.ucsc.edu/) (24). Primers used for bisulfite sequencing PCR were designed with the aid of Methprimer (25) (Table I). A 50-μl PCR mixture contained 10 mM dNTP, 1.8 μl of each 10 pmol primers, 1 U of Phusion U Hot Start DNA polymerase (Thermo Scientific, #F-555S), 10 μl of GC buffer, 1 μl of DMSO, and 4 μl of bisulfite-treated DNA.

PCR program with the following thermal settings was applied: hot start at 98°C for 30 sec, 30 cycles of denaturation at 98°C for 10 sec, annealing temperature for 30 sec (Table I), extension at 72°C for 30 sec, and a final extension at 72°C for 5 min. The PCR products were sent directly to Bioneer (South Korea) for purification and Sanger sequencing. The sequencing was performed one time with forward primers, and the results were analyzed using BioEdit V 7.2.6.1.

**Table 1:** Primers information for amplification of *amh* and *kitl* genes


**Gene**	**Primer sequence (5'-3')**	**Annealing temperature (°C)**	**Accession no.**	**Primer position**
*amh*	Forward: GGAGATGGGAGTTAYTYAAGGA	56	NM_007445	–225, –204
	Reverse: CAAACTRTAACACARCCCCCAT			68, 48
*kitl*	Forward: AAATTAGTTAAGAYAATGGGATTG	54	NM_013598	–465, –442
	Reverse: AACAACCTRCAATARCTRCCCT			–243, –264
Y = C or T, R = A or G				

### Ethical considerations

Animal studies were carried out following the ethical principles of the Declaration of Helsinki at the Damghan University in 2018. All experimental procedures were approved by the Animal Care Center of Damghan University (No: 35: 2018).

### Statistical analysis

Data were analyzed using SPSS software, version 21 (SPSS Inc., Chicago, IL, USA). The Shapiro–Wilk test was performed for evaluating the normality distribution of variables. Due to the non-normal distribution of data, the Kruskal–Wallis test was used to determine the significance of changes in the follicle diameter. To compare the mean of follicular degeneration and antral cavity formation, one-way ANOVA and Tukey's test were used. A p-value < 0.05 was considered as statistical significance.

## 3. Results

### Effect of BPA on follicle growth and survival

At the beginning of the culture, the mean follicle diameter was 135.06 ± 13.30 μm, and no significant difference was observed among them (p = 0.34). Morphological changes of the follicles were analyzed every other day during the culture periods. On the second day of the culture, preantral follicles were attached to the bottom of the dishes. The diameter of follicles increased gradually, and on the eight day of the culture reached 408.66 ± 13.38 μm, with a nearly three-fold increase. The follicular size was measured after adding ethanol and BPA concentrations in the experimental groups (Table II). By day 9 of the culture, the diameter of follicles exposed to ethanol for 24 hr was observed to be significantly higher than those exposed to 50 μM and 100 μM of BPA (p = 0.00; Table II). Also, the size of follicles in the presence of 50 μM and 100 μM of BPA for 72 hr enhanced. However, exposure to 100 μM of BPA significantly decreased the size of follicles in comparison to those exposed to 50 μM of BPA during the same time (p = 0.00; Table II). Furthermore, on day 11 of the culture, there was only a significant difference between the diameter of follicles cultured with 100 μM of BPA and ethanol solvent control (p = 0.00; Table II).

Table III lists the comparison of the incidence of follicular degeneration and antral cavity formation in the presence of BPA concentrations and solvent. Degenerated follicles were identified by a dark oocyte, the single layer of granulosa cells, or fragmentation signs in follicle (26). Exposure to BPA (50 and 100 μM) did not significantly influence survival and antral cavity formation in follicles in comparison with the ethanol solvent group (p = 0.41; Table III).

### Effect of BPA on DNA methylation

**Figure 1:**
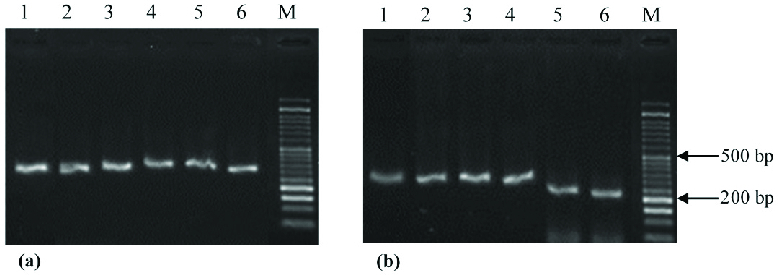
The electrophoresis of *amh* (a) and *kitl* (b) PCR products. Lane M, DNA molecular weight marker (DM1100, SMOBIO Technology); the PCR products of lane 1–6 were amplified from granolusa cells in the following groups: ethanol-solvent control for 24 hr, ethanol-solvent control for 72 hr, 50 μM of BPA for 24 hr, 50 μM of BPA for 72 hr, 100 μM of BPA for 24 hr, and 100 μM of BPA for 72 hr.

PCR products of 292 and 223 bp were amplified from *amh* and *kitl *promoters (Figure 1). Sequencing revealed that three CpG sites in the amplified regions of the *amh* promoter were partially methylated in solvent control. The percentage of methylated cytosine was calculated according to the peak heights and the following formula: % mC = [C/(C + T)] × 100 (27) (Table IV). The level of methylated cytosines was reduced in the presence of BPA concentrations, and 100 μM of BPA had a higher demethylation effect than 50 μM of BPA (Table IV). Seven CpG sites were detected in the PCR product of *kitl *promoter, and all of the cytosines were unmethylated in the experimental groups.

**Table 2:** Diameter of follicles in the presence of BPA concentrations


**Groups**	**9${}^{\mathrm{𝐭𝐡}}$ day (after 24 hr treatment)**		**11${}^{\mathrm{𝐭𝐡}}$ day (after 72 hr treatment)**	
	**Total number of follicles**	**Follicle diameter (μm)**	**Total number of follicles**	**Follicle diameter (μm)**
**Ethanol-solvent control**	50	440.03 ± 23.77	50	484.10 ± 18.56
**50 μM of BPA**	50	414.23 ± 11.14*	110	474.31 ± 23.74
**100 μM of BPA**	50	413.43 ± 12.45*	110	429.56 ± 10.38*
Data presented as Mean ± SD.${}^{*}$ p ≤ 0.001, BPA: Bisphenol A				

**Table 3:** Effect of BPA on degeneration and antral cavity formation in follicles


**Groups**	**Total number of follicles**	**Degenerated number **	**Antrum formation number **
**Ethanol-solvent control for 24 hr**	50	6 (12.00 ± 0.0)	43 (86.00 ± 2.51)
**Ethanol-solvent control for 72 hr**	50	6 (12.00 ± 0.0)	43 (86.00 ± 2.51)
**50 μM of BPA for 24 hr**	50	7 (14.00 ± 1.0)	42 (84.00 ± 4.04)
**50 μM of BPA for 72 hr**	110	23 (20.90 ± 1.70)	85 (77.27 ± 3.59)
**100 μM of BPA for 24 hr**	50	9 (18.00 ± 1.73)	40 (80.00 ± 3.60)
**100 μM of BPA for 72 hr**	110	27 (24.54 ± 2.87)	82 (74.54 ± 2.64)
Data are expressed as number (percentage ± standard deviation), BPA: Bisphenol A			

**Table 4:** Percentage of methylated cytosine in the promoter region of *amh* in the presence of BPA


**Groups**		**CpG sites (%)**		
**1${}^{\mathrm{𝐬𝐭}}$ CpG**	**2${}^{\mathrm{𝐧𝐝}}$ CpG**	**3${}^{\mathrm{𝐫𝐝}}$ CpG**
**Ethanol-solvent control for 24 hr **	87.37	69.50	85.36
**Ethanol-solvent control for 72 hr**	88.23	70.15	87.89
**50 μM of BPA for 24 hr**	82.22	25.00	81.63
**50 μM of BPA for 72 hr**	84.74	50.00	82.14
**100 μM of BPA for 24 hr**	75.60	12.50	70.00
**100 μM of BPA for 72 hr**	74.28	31.03	76.58
BPA: Bisphenol A			

## 4. Discussion

Chemical agents are part of our modern life, and long-term exposure to their toxic effects may compromise human health. Several studies have shown that BPA has inappropriate effects on the female reproductive system of humans and animals (5, 6, 9, 28). Ovarian follicles are the basic female reproductive units, and the endocrine system is vital for their function (13). Since follicular granulosa cells express estrogen receptors, they could be a candidate target for the BPA effect (21, 29).

In the current study, our results showed that exposure to 100 μM of BPA had an inhibitory effect on mouse follicles growth through 72-hr culture, while under experimental conditions, BPA did not significantly change the percentage of degeneration or antrum formation in follicles. Peretz and co-workers in two separate studies, observed that BPA at concentrations of 440 μM and 100 μg/ml have an inhibitory effect on cultured murine antral follicles (19, 30). Furthermore, BPA at 100 μg/ml increased follicle degeneration after 48 hr (19). Peretz and co-authors. suggested that BPA, by interfering with steroidogenesis, decreases follicular growth (30). In another study, Ziv-Gal *et al*. showed that 110–438 μM of BPA inhibits the growth of mouse antral follicles (18). Similar results demonstrated by Wang and colleagues showed that 45 μM BPA significantly reduced the growth of preantral mouse follicles, and also disrupted normal follicle development through the reduction of antral follicle formation (20). However, the lower concentration of BPA (3-30 μM) did not have a significant influence on mouse preantral follicles growth and antral cavity formation (31). Although the negative effect of a high concentration of BPA on cell growth was reported in the aforementioned studies, the results are not comparable because of the differences in follicle stage, mouse strain, and period of culture time.

There are some evidences that in addition to growth inhibition, BPA disrupts cytoskeleton (32) and spindle formation (32, 33), induces apoptosis (21, 32) and epigenetic effects on histone and DNA methylation (32). The results of the present study showed that the seven CpG sites examined in the promoter region of *kitl *were unmethylated, and BPA under any experimental conditions had no effect on the level of methylation. In the case of the *amh* promoter region, three CpG sites were partially methylated before BPA treatment. In the presence of BPA, the partially methylated pattern was observed again, however, BPA at 100 μM concentration profoundly reduced the percentage of methylation. Partial methylation pattern displays incomplete bisulfite conversion or partial methylation in the original DNA template (34). According to the applied thermal protocol with >99% efficiency, it seems that the studied promoter region of *amh* was in partial methylation state.

In line with our findings, some researches indicated that BPA reduces the percentage of 5mC in methylated DNA. The experiment of Chao and colleagues illustrated that hypodermic injection of BPA into mice reduces the methylation level of imprinted genes during oogenesis (33). In another study, the exposure of zebrafish with 1 mg/L BPA for 15 days significantly decreased global DNA methylation in ovaries. The reduction of DNA methyltransferase (*dnmt1*) expression was in association with the hypomethylation of DNA (9). Wang and co-authors showed that BPA at a concentration of 250 μM decreased 5mC level and *dnmt3b* expression in porcine oocytes (32). However, in Zhang *et al* study, both 10 and 100 μM BPA increased the percentage of 5mC in 3'UTR of *Lhx8* (transcription factor) in mouse oocytes by preventing DNA demethylation (35). In another research, prolonged exposure of mouse follicles to 3 nM of BPA caused abnormal methylation patterns of maternally imprinted genes in oocytes (36). As it is clear, BPA induces epigenetic alterations in the female reproductive tract, and this can be accounted as one of the vital reasons for its reproductive toxicity.

## 5. Conclusion

The observations of the current study indicate the inhibitory effect of BPA on follicle growth under in vitro culture conditions. Furthermore, BPA reduces methylation level of *amh* promoter in granulosa cells. While AMH has a significant role in early follicular growth, it seems that modification of its methylation status may lead to gene expression alteration. More studies need to investigate various aspects of the epigenetic impact of BPA on the female reproductive system.

##  Conflict of Interest

The authors declare that there is no conflict of interest.

## References

[1] Maqbool F, Mostafalou S, Bahadar H, Abdollahi M. Review of endocrine disorders associated with environmental toxicants and possible involved mechanisms. *Life Sci* 2016; 145: 265–273. 10.1016/j.lfs.2015.10.022 26497928

[2] Swedenborg E, Rüegg J, Makela S, Pongratz I. Endocrine disruptive chemicals: Mechanisms of action and involvement in metabolic disorders. *J Mol Endocrinol *2009; 43: 1–10. 10.1677/JME-08-0132 19211731

[3] Kundakovic M, Champagne FA. Epigenetic perspective on the developmental effects of bisphenol A. *Brain Behav Immun* 2011; 25: 1084–1093. 10.1016/j.bbi.2011.02.005 PMC3703316 21333735

[4] Matuszczak E, Komarowska MD, Debek W, Hermanowicz A. The impact of bisphenol A on fertility, reproductive system, and development: A review of the literature. *Int J Endocrinol* 2019; 2019: 4068717. 10.1155/2019/4068717 PMC6481157 31093279

[5] Chianese R, Troisi J, Richards S, Scafuro M, Fasano S, Guida M, et al. Bisphenol A in reproduction: Epigenetic effects. *Curr Med Chem* 2018, 25: 748–770. 10.2174/0929867324666171009121001 28990514

[6] Huo X, Chen D, He Y, Zhu W, Zhou W, Zhang J. Bisphenol-A and female infertility: A possible role of gene-environment interactions. *Int J Environ Res Public Health* 2015; 12: 11101–11116. 10.3390/ijerph120911101 PMC4586663 26371021

[7] Mansur A, Israel A, Combelles CMH, Adir M, Racowsky C, Hauser R, et al. Bisphenol-A exposure and gene expression in human luteinized membrana granulosa cells in vitro. *Hum Reprod* 2017; 32: 409–417. 10.1093/humrep/dew316 PMC6260419 27979917

[8] Ideta-Otsuka M, Igarashi K, Narita M, Hirabayashi Y. Epigenetic toxicity of environmental chemicals upon exposure during development - Bisphenol A and valproic acid may have epigenetic effects. *Food Chem Toxicol* 2017; 109: 812–816. 10.1016/j.fct.2017.09.014 28888737

[9] Laing LV, Viana J, Dempster EL, Trznadel M, Trunkfield LA, Uren Webster TM, et al. Bisphenol A causes reproductive toxicity, decreases dnmt1 transcription, and reduces global DNA methylation in breeding zebrafish (*Danio rerio*). *Epigenetics *2016, 11: 526–538. 10.1080/15592294.2016.1182272 PMC4939919 27120497

[10] Ruiz-Hernandez A, Kuo CC, Rentero-Garrido P, Tang WY, Redon J, Ordovas JM, et al. Environmental chemicals and DNA methylation in adults: A systematic review of the epidemiologic evidence. *Clin Epigenetics* 2015; 7: 55. 10.1186/s13148-015-0055-7 PMC4433069 25984247

[11] Jiang JY, Xiong H, Cao M, Xia X, Sirard MA, Tsang BK. Mural granulosa cell gene expression associated with oocyte developmental competence. *J Ovarian Res* 2010; 3: 6. 10.1186/1757-2215-3-6 PMC2845131 20205929

[12] Cuiling L, Wei Y, Zhaoyuan H, Yixun L. Granulosa cell proliferation differentiation and its role in follicular development. *Chin Sci Bull* 2005; 50: 2665–2671.

[13] Grasselli F, Baratta L, Baioni L, Bussolati S, Ramoni R, Grolli S, et al. Bisphenol A disrupts granulosa cell function. *Domest Anim Endocrinol* 2010; 39: 34–39. 10.1016/j.domaniend.2010.01.004 20172683

[14] Kidder GM, Vanderhyden BC. Bidirectional communication between oocytes and follicle cells: Ensuring oocyte developmental competence. *Can J Physiol Pharmacol* 2010; 88: 399–413. 10.1139/y10-009 PMC3025001 20555408

[15] Driancourt M, Reynaud K, Cortvrindt R, Smitz J. Roles of KIT and KIT ligand in ovarian function. *Rev Reprod *2000; 5: 143–152. 10.1530/ror.0.0050143 11006164

[16] van Houten ELAF, Themmen APN, Visser JA. Anti-Müllerian hormone (AMH): Regulator and marker of ovarian function. *Ann Endocrinol* 2010; 71: 191–197. 10.1016/j.ando.2010.02.016 20362961

[17] Knight PG, Glister C. TGF-β superfamily members and ovarian follicle development. *Reproduction* 2006; 132: 191–206. 10.1530/rep.1.01074 16885529

[18] Ziv-Gal A, Craig ZR, Wang W, Flaws JA. Bisphenol A inhibits cultured mouse ovarian follicle growth partially via the aryl hydrocarbon receptor signaling pathway. *Reprod Toxicol *2013; 42: 58–67. 10.1016/j.reprotox.2013.07.022 PMC3836856 23928317

[19] Peretz J, Craig ZR, Flaws JA. Bisphenol A inhibits follicle growth and induces atresia in cultured mouse antral follicles independently of the genomic estrogenic pathway. *Biol Reprod *2012; 87: 63–74. 10.1095/biolreprod.112.101899 PMC3464906 22743301

[20] Wang X, Jiang SW, Wang L, Sun Y, Xu F, He H, et al. Interfering effects of bisphenol A on in vitro growth of preantral follicles and maturation of oocyes. *Clin Chim Acta* 2018; 485: 119–125. 10.1016/j.cca.2018.06.041 29958887

[21] Xu J, Osuga Y, Yano T, Morita Y, Tang X, Fujiwara T, et al. Bisphenol A induces apoptosis and G2-to-M arrest of ovarian granulosa cells. *Biochem Biophys Res Commun *2002; 292: 456–462. 10.1006/bbrc.2002.6644 11906184

[22] Council NR. Guide for the care and use of laboratory animals. 8${}^{\mathrm{th}}$ Ed. Washington, DC: The National Academies Press, 2011. 21595115

[23] Vafere Koohestani N, Zavareh S, Lashkarbolouki T, Azimipour F. Exposure to cell phone induce oxidative stress in mice preantral follicles during in vitro cultivation: An experimental study. *Int J Reprod Biomed* 2019; 17: 637–646. 10.18502/ijrm.v17i9.5099 PMC6804329 31646258

[24] Karolchik D, Hinrichs AS, Furey TS, Roskin KM, Sugnet CW, Haussler D, et al. The UCSC Table Browser data retrieval tool. *Nucleic Acids Res* 2004; 32: 493–496. 10.1093/nar/gkh103 PMC308837 14681465

[25] LC L-Ch, Dahiya R. MethPrimer: Designing primers for methylation PCRs. *Bioinformatics* 2002; 18: 1427–1431. 10.1093/bioinformatics/18.11.1427 12424112

[26] Uslu B, Dioguardi CC, Haynes M, Miao DQ, Kurus M, Hoffman G, et al. Quantifying growing versus non-growing ovarian follicles in the mouse. *J Ovarian Res* 2017; 10: 3–13. 10.1186/s13048-016-0296-x PMC5237173 28086947

[27] Parrish RR, Day JJ, Lubin FD. Direct bisulfite sequencing for examination of DNA methylation with gene and nucleotide resolution from brain tissues. *Curr Protoc Neurosci* 2012; 7: 24. 10.1002/0471142301.ns0724s60 PMC3395468 22752896

[28] Caserta D, Di Segni N, Mallozzi M, Giovanale V, Mantovani A, Marci R, et al. Bisphenol A and the female reproductive tract: An overview of recent laboratory evidence and epidemiological studies. *Reprod Biol Endocrinol* 2014; 12: 37. 10.1186/1477-7827-12-37 PMC4019948 24886252

[29] Drummond AE, Fuller PJ. Ovarian actions of estrogen receptor-β: An update. *Semin Reprod Med* 2012; 30: 32–38. 10.1055/s-0031-1299595 22271292

[30] Peretz J, Gupta RK, Singh J, Hernández-Ochoa I, Flaws JA. Bisphenol A impairs follicle growth, inhibits steroidogenesis, and downregulates rate-limiting enzymes in the estradiol biosynthesis pathway. *Toxicol Sci *2011; 119: 209–217. 10.1093/toxsci/kfq319 PMC3003833 20956811

[31] Lenie S, Cortvrindt R, Eichenlaub-Ritter U, Smitz J. Continuous exposure to bisphenol A during in vitro follicular development induces meiotic abnormalities. *Mutat Res *2008; 651: 71–81. 10.1016/j.mrgentox.2007.10.017 18093867

[32] Wang T, Han J, Duan X, Xiong B, Cui XS, Kim NH, et al. The toxic effects and possible mechanisms of Bisphenol A on oocyte maturation of porcine in vitro. *Oncotarget* 2016; 7: 32554–32565. 10.18632/oncotarget.8689 PMC5078033 27086915

[33] Chao HH, Zhang XF, Chen B, Pan B, Zhang LJ, Li L, et al. Bisphenol A exposure modifies methylation of imprinted genes in mouse oocytes via the estrogen receptor signaling pathway. *Histochem Cell Biol* 2012; 137: 249–259. 10.1007/s00418-011-0894-z 22131059

[34] Li Y, Tollefsbol TO. DNA methylation detection: Bisulfite genomic sequencing analysis. *Methods Mol Biol* 2011; 791: 11–21. 10.1007/978-1-61779-316-5_2 PMC3233226 21913068

[35] Zhang T, Li L, Qin XS, Zhou Y, Zhang XF, Wang LQ, et al. Di-(2-ethylhexyl) phthalate and bisphenol A exposure impairs mouse primordial follicle assembly in vitro.* Environ Mol Mutagen *2014; 55: 343–353. 10.1002/em.21847 24458533

[36] Trapphoff T, Heiligentag M, El Hajj N, Haaf T, Eichenlaub-Ritter U. Chronic exposure to a low concentration of bisphenol A during follicle culture affects the epigenetic status of germinal vesicles and metaphase II oocytes. *Fertil Steril* 2013; 100: 1758– 1767. 10.1016/j.fertnstert.2013.08.021 24034936

